# Parietal low beta rhythm provides a dynamical substrate for a working memory buffer

**DOI:** 10.1073/pnas.1902305116

**Published:** 2019-08-01

**Authors:** Alexandros Gelastopoulos, Miles A. Whittington, Nancy J. Kopell

**Affiliations:** ^a^Department of Mathematics and Statistics, Boston University, Boston, MA 02215;; ^b^Centre for Neuroscience, University of York, York YO10 5DD, United Kingdom

**Keywords:** cell assemblies, biophysical model, frontoparietal coordination

## Abstract

Working memory (WM) is a component of the brain’s memory systems vital for interpretation of sequential sensory inputs and consequent decision making. Anatomically, WM is highly distributed over the prefrontal cortex (PFC) and the parietal cortex (PC). Here we present a biophysically detailed dynamical systems model for a WM buffer situated in the PC, making use of dynamical properties believed to be unique to this area. We show that the natural beta1 rhythm (12 to 20 Hz) of the PC provides a substrate for an episodic buffer that can synergistically combine executive commands (e.g., from PFC) and multimodal information into a flexible and updatable representation of recent sensory inputs. This representation is sensitive to distractors, it allows for a readout mechanism, and it can be readily terminated by executive input. The model provides a demonstration of how information can be usefully stored in the temporal patterns of activity in a neuronal network rather than just synaptic weights between the neurons in that network.

## Significance

Working memory (WM) is believed to consist of several components, one of which is an episodic buffer, whose task is to combine sensory stimuli with executive commands into flexible, task-relevant representations and hold them briefly for later use. Little is known about where in the brain the episodic buffer lies or about its underlying physiology. We propose that physiological properties of the parietal cortex, which can produce a unique brain rhythm at low beta (12 to 20 Hz), make this cortex an excellent candidate for a substrate of such a buffer. We show that cell assemblies shaped by this rhythm have the required properties of a WM buffer, allowing for robust yet manipulable representations of sensory stimuli.

Working memory (WM) is a limited-capacity, short-lasting store for recent inputs (several seconds only) but distinct from short-term memory owing to its ability to allow rehearsal and manipulation (updating) of contents (1). It is also seen as distinct from more long-term memory stores not just on the basis of its duration but also on its ability to be actively terminated on task shifting (2, 3). There are many theories considering the structure and operation of WM (4) with perhaps the most successful and long-lasting of these remaining faithful to the ideas originally proposed by Baddeley (e.g., ref. 5, updated in refs. 6 and 7). Within this framework, WM is seen as having 4 core components: a central executive, 2 slave systems specifically responsible for representing visuospatial and auditory (language) information, and an episodic buffer to synergistically combine both executive commands and multimodal information into a temporary, manipulable representation of recent sensory inputs relevant to the currently attended task.

Assigning actual brain regions to the above theoretical components is not straightforward. However, there is good evidence for the central executive residing in the prefrontal cortex. Using functional imaging, prefrontal cortex in general has been shown to be vital for WM (8), and further studies specifically implicate the dorsolateral prefrontal (9) and the anterior cingulate cortices (10). Other studies have also implicated the inferior frontal gyrus in WM performance (11). The visuospatial and auditory slaves appear to correspond to regions primarily involved in processing these modalities of information. For example, spatial corresponds to premotor cortex (12), and active speech corresponds to Broca’s area (13).

An anatomical substrate for the episodic buffer is harder to pin down. The region must be able to interact functionally with both the frontal central executive and the more distributed slave systems. Perhaps the most promising regions all lie in parietal cortex: Interactions between frontal and parietal cortices are vital for WM function (14–16). Activity in superior parietal areas has been actively correlated with WM outcome (11), maintenance (1), and manipulation (17). The parietal cortex has strong reciprocal connectivity to many prefrontal regions and is a convergence point for multiple modes of sensory input (18, 19). In addition, the episodic buffer also needs to act as an interface with long-term memory systems, and evidence suggests that the parietal cortex does so, at least for inhibitory avoidance tasks (20).

The parietal cortex is also an attractive substrate for the episodic buffer from a neuronal dynamics perspective. Baddeley’s view of this component of WM made clear the exceptional demands on such a region, with connections to multiple different cortical systems, each potentially with different temporal codes for the information held. Some regions of prefrontal cortex appear to demonstrate flexible temporal structures (21) that might subserve such demands. However, the multidimensional code envisaged by Baddeley—where each dimension constitutes the cortical activity representing a modality-specific component of the sensory experience being held in the buffer—is perhaps best demonstrated by the beta1 (12 to 18 Hz) frequency rhythm as manifest in parietal cortex (22, 23). Owing to the seemingly unique behavior of layer 5 intrinsic bursting neurons in this region (24), beta1 rhythms can occur as the concatenation sum of layer 5 beta2 rhythm (20 to 30 Hz) and superficial layer low-gamma rhythms (30 to 50 Hz). Both of these rhythms have been implicated in various aspects of WM (25–27). This ability to combine different rhythms through concatenation leads to a rich and complex dynamic in parietal cortex that is predicted to act as a flexible substrate for short-term memories (28).

We generated a number of computational models of parietal beta1 frequency activity to explore its ability to act as a substrate for the episodic buffer. We demonstrate that this pattern of network dynamics satisfies all of the relevant proposed properties: It represents a flexible memory substrate for previously active neuronal assemblies in a manner controlled by top-down, central executive inputs and requires continuous rehearsal owing to its dynamic (rather than hard-wired) nature. It can incorporate relevant new sensory inputs while itself remaining robust, it can maintain efficiency by rejecting weak components, and it can be terminated by executive input when no longer required or by distractors—bottom-up irrelevant sensory input. Our computational models are based on the parietal beta1 model introduced in ref. 23. The same model was used to study representation and interaction of novel and familiar stimuli in ref. 28. See *Discussion* for a comparison of the results of the current paper to ref. 28.

## Results

### Basic Properties of the Parietal beta1 Rhythm Relevant to WM.

**Fig. 1.**
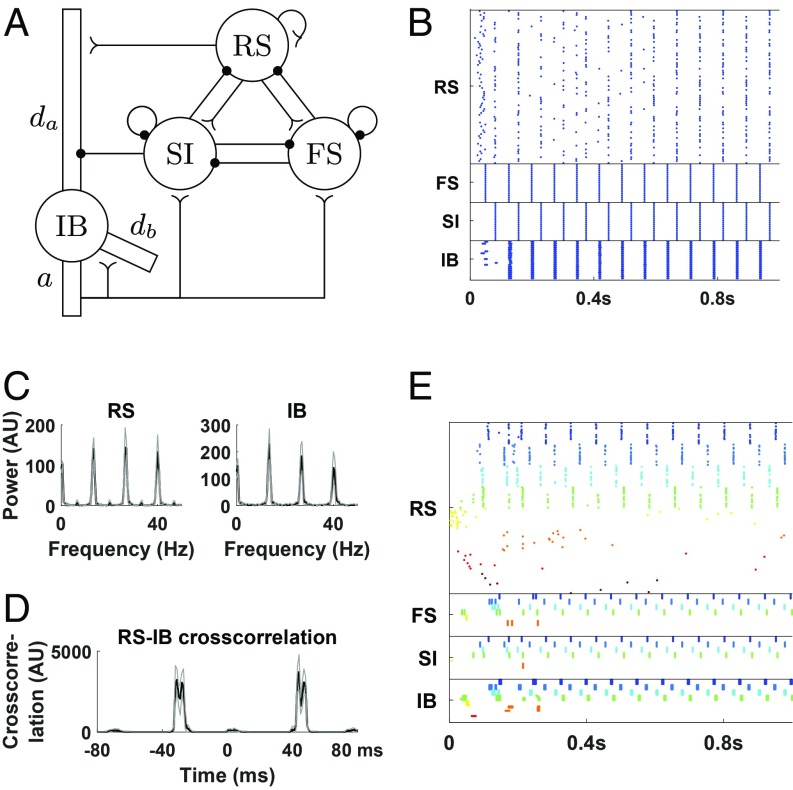
The beta1 network model. (*A*) The network. Each circle represents a population of cells of the same type. There are 80 RS cells and 20 cells of each of FS, SI (LTS in ref. 23), and IB, with connectivity as shown in the picture; reverse arrowheads denote excitatory synapses, while filled circles denote inhibitory synapses (also see *Materials and Methods*). $d_{a}$, apical dendrite; $d_{b}$, basal dendrite; a, axon. Modified from ref. 23, which is licensed under CC BY 3.0. (*B*) Rastergram of a simulation of the network in *A*. Each line corresponds to a single cell with each dot corresponding to a spike. Both superficial and deep layers exhibit a beta1 rhythm but out of phase. (*C*) Power spectra of RS and IB cells. Note that peaks around 27 and 40 Hz are harmonics of the beta1 band peak (13 to 14 Hz), but in the case of RS cells they are accentuated because of some extra firing in between cycles. (*D*) Cross-correlogram of RS and IB spikes. IB cells tend to fire around 40 ms after and 30 ms before RS cells. (*E*) We include 8 columns, each with the same characteristics as in *B* (but with 1/4 of the cells) and represented by a different color, except that the last 4 columns are less excitable, modeling the fact that they have not received any prior sensory input. There are weak connections between pyramidal cells of both deep and superficial layers of different columns (*Materials and Methods*), but they do not result in activation of the less excitable columns, although noise induces some minimal activity in them. Black lines in *C* and *D* are average of 10 simulations. Gray lines indicate ±1SD. AU, arbitrary units.

WM is considered mechanistically distinct from other forms of memory owing to its relative lack of reliance on (short-term) changes in synaptic weights and (long-term) remodeling of neuronal connectivity. Without these well-documented substrates for memory, how can an engram be maintained? We propose that maintenance is a direct consequence of the rehearsal properties of WM, i.e., continuous activity in the neurons involved. In central executive regions, this is thought to be achieved by persistent firing of prefrontal neurons (29). However, this is a rather inflexible process unlikely to fulfill the proposed role of the episodic buffer (7). A more complex form of persistent activity has been reported in PC beta1 rhythms in vitro. Following a brief period of excitation, resulting gamma and beta2 frequency activity persists but exhibits a concatenation-of-periods pattern, where 1 period of a local cortical beta1 rhythm is formed as a sum of superficial layer gamma (25 ms) and deep layer beta2 (40 ms) rhythms, with outputs from neurons in one layer iteratively driving outputs from the other (22). Specifically, fast-spiking (FS) interneurons are activated by deep layer pyramidal cells (intrinsically bursting [IB] cells) and inhibit superficial layer pyramidal cells (regular spiking [RS] cells). The latter fire by rebound from inhibition about 25 ms later and activate slow inhibitory (SI) cells, which in turn inhibit the deep layer IB cells. The IB cells fire by rebound from inhibition about 40 ms later, starting a new cycle. Once this activity is initiated, very little drive is required to maintain it; instead of continuous excitation, it is time-delayed responses to inhibition from cells within the network that maintain it (in vitro the reduction of excitation is modeled by use of glutamate antagonists). Within this dynamic framework multiple assemblies of neurons, temporally coded by gamma rhythms, can coexist within the concatenated rhythm. We use a modified version of the Kramer et al. (23) model (Fig. 1*A*; also see *Materials and Methods*) as the substrate for episodic buffer activity following activation in individual cortical minicolumns (MCs; referred to as columns in ref. 23) throughout.

The relevant model behavior can be summarized as follows: An initial, transient presentation of bottom-up sensory input to selected MCs enhances synaptic connectivity in the deep layers, which allows the formation of a persistent beta1 rhythm when subsequently much of this excitation has faded. As explained above, this persistent beta1 activity is maintained by iterative, reciprocal excitation of deep and superficial layers, resulting in the concatenation of gamma (ca. 30 ms) and beta2 (ca. 40 ms) network time constants to form a third, beta1 (ca. 70 ms) period (Fig. 1 *B*–*D*). This activity is stimulus specific as MCs that do not receive any input remain silent (Fig. 1*E*). It has its own dynamic signature (the beta1 frequency) but maintains time constants appropriate for processing both further bottom-up (gamma) inputs and top-down executive inputs (beta2)—properties inherent in the current view of episodic buffer function (see the *Introduction*). This continuing activity, in the absence of further sensory input, maintains the memory in the buffer.

### Top-Down Excitatory Input to Parietal Columns Can Create Cell Assemblies.

The above dynamic substrate for the episodic buffer is generated at the level of single parietal cortical MCs following a single episode of sensory excitation. However, the buffer must be able to combine multiple echoes of such prior excitation to form a useful representation of polymodal sensory input. This is proposed to occur under prefrontal, central executive (top-down) control. Prefrontal cortex, which provides top-down input to the parietal cortex, exhibits both beta and gamma rhythms (30, 31), with top-down information in general seen to use the alpha–beta frequency bands (32, 33). We thus considered the effect of an input to the deep layer neurons, for rhythmic input of various frequencies, as well as tonic input.

**Fig. 2.**
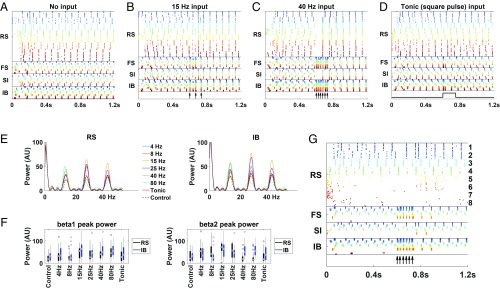
Top-down input to the deep layers synchronizes beta1 columns. (*A*) We include 8 copies of our original beta1 column, each with the same characteristics as in Fig. 1 and represented by a different color. There are weak connections between pyramidal cells of both deep and superficial layers of different columns (*Materials and Methods*). (*B* and *C*) The same brief input is given to the deep layers of each of the columns, of frequency (*B*) 15 Hz or (*C*) 40 Hz. The input is in the form of brief injections of current that resemble synaptic currents (*Materials and Methods*), at the times indicated by black arrows. (*D*) Same as *B* and *C* but with tonic input in the form of a square pulse (black trace). (*E*) Power spectrogram after termination of the input presentation (900 to 1,200 ms). In the cases of 4 and 8 Hz the input duration was 300 ms; in all other cases it was 150 ms. (*F*) Boxplots of beta1 (*Left*) and beta2 (*Right*) peak power after input presentation (900 to 1,200 ms) for RS and IB cells. Beta1 power peak is significantly higher compared with control when the input is rhythmic of frequency 15, 40, or 80 Hz. Beta2 power peak is significantly higher compared with control when the input is 15 or 25 Hz and weakly significantly higher in the case of the RS cells for 4 or 8 Hz input. *P* values (1-sided Wilcoxon rank-sum test, *n* = 20) for beta1 are as follows: 4 Hz, 8 Hz, and tonic input, RS and IB cells, p>0.05; 15 Hz, $p_{RS}=0.0053$, $p_{IB}=0.0062$; 25 Hz, $p_{RS}=0.0481$, $p_{IB}=0.0568$; 40 Hz, $p_{RS}=0.0077$, $p_{IB}=0.0028$; 80 Hz, $p_{RS}=0.0005$, $p_{IB}=0.0014$. *P*-values for beta2 are as follows: 4 Hz, $p_{RS}=0.0455$, $p_{IB}=0.0568$; 8 Hz, $p_{RS}=0.0382$, $p_{IB}=0.2047$; 15 Hz, $p_{RS}=0.0008$, $p_{IB}=0.001$; 25 Hz, $p_{RS}=0.0128$, $p_{IB}=0.012$; 40 Hz, 80 Hz, and tonic input, RS and IB cells, p>0.05. (*G*) Same as *A*–*D* but the last 4 columns are less excitable (*SI Appendix*), and we give a 40-Hz input to the deep layers of columns 1, 2, 5, and 6. Columns that neither are active at beta1 nor receive the top-down input (columns 7 and 8) remain silent. Columns that are not active at beta1 but receive the top-down input (columns 5 and 6) are active only while the top-down input lasts. For clarity, columns are numbered only for RS cells.

We included 8 copies of MCs generating the poststimulus, concatenated beta1 rhythm. The MCs were weakly connected with each other through both deep and superficial layers in a manner that maintained individual MC beta1 rhythms but generated only weak inter-MC synchrony (Fig. 2*A*). After a baseline initial time interval, we gave the same top-down input to all MCs, through the deep layers, lasting about 150 ms (34, 35) (300 ms in the cases of 4 and 8 Hz input). Rhythmic input at sufficiently high frequency (beta1 or larger), but not lower frequency or tonic input, was able to synchronize all 8 MCs, and this imposed temporal order lasted long after the input ceased and was reflected in the combined power in the beta1 band (Fig. 2 *B*–*F*). Interestingly, in the case of beta1 and beta2 frequency input, after the input terminated, there was often an increase in the beta2 power, around 27 Hz (Fig. 2 *E* and *F*). The reason was that although each MC was oscillating at beta1, some of the MCs were antiphase with the rest, creating a rhythm of double the frequency. This was due to the dynamics of the individual MCs; the phase of each MC immediately after the input termination was determined by whether the RS or IB cells would fire first, which appeared to be random (Fig. 2*B*). The duration of the input could be made shorter than 150 ms and still get the synchrony, but a single brief pulse was not enough to synchronize the MCs (*SI Appendix*, Fig. S1). MCs that were not contributing to the established beta1 frequency activity (presumably because they had not received prior bottom-up input) did not alter their ongoing activity patterns beyond the duration of the subsequent top-down input (Fig. 2*G*).

### Cell Assemblies, Active at beta1 Frequency, Can Be Manipulated by Addition.

An important property of WM is the ability to manipulate its content by adding and subtracting elements (MCs). Using an already established beta1 rhythm on a set of cortical MCs, we first model the process of inclusion of additional MCs. In particular, we consider a learned pattern of activation in which there is a sequence of stimuli activating a sequence of MCs; the model shows how the beta1 rhythm fosters the addition of new MCs associated with ongoing stimuli.

**Fig. 3.**
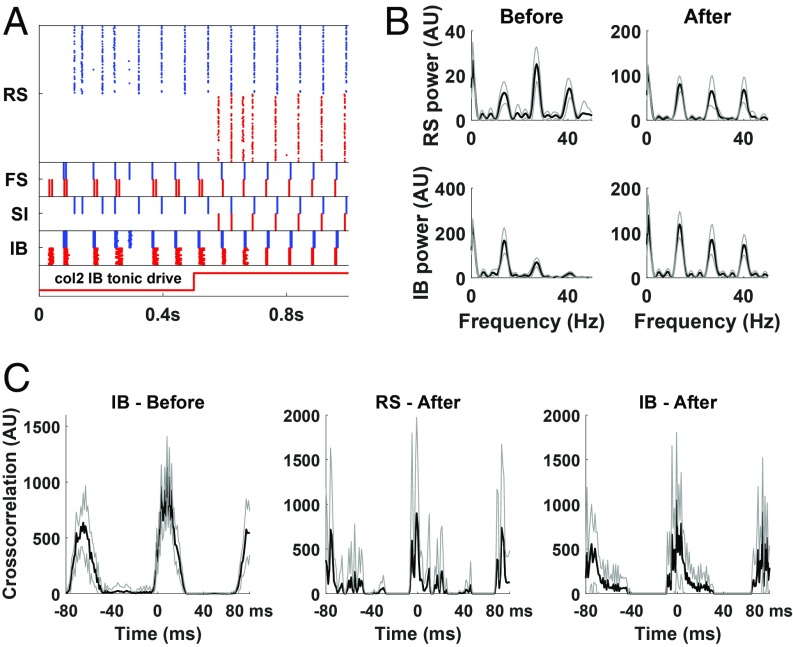
Simulation of a network of 2 unidirectionally connected columns. Each column is as in Fig. 1, except that RS tonic current is initially lower for column 2 and increased to normal value at around 500 ms (*Materials and Methods*). IB cells of column 1 synapse onto IB cells of column 2. (*A*) Rastergram. Cells in the 2 columns are distinguished by blue (column 1) and red colors (column 2). Initially, pyramidal cells only in the deep layers of the 2 columns are synchronized, while the RS cells of column 2 are silent. When the second column is fully activated and after a transient interval, the entire columns coordinate. (*B*) Power spectra of RS and IB cells (both columns) before (200 to 500 ms) and after (700 to 1,000 ms) the increase in column 2 RS tonic drive. Beta1 power of RS cells is 5.27 times higher ($p=1.8\cdot 10^{-4}$, 2-sided Wilcoxon rank-sum test, *n* = 10), and beta1 power of IB cells is 31% lower ($p=1.8\cdot 10^{-4}$) in the after condition. (*C*) Cross-correlation of RS/IB cells with the corresponding cells of the other column, before (only for IB) and after the increase in column 2 RS tonic drive. Black lines in *B* and *C* are average of 10 simulations. Gray lines are ±1SD.

We consider a network consisting of 2 MCs, with the IB cells of MC1 effectively exciting IB cells of MC2 but not vice versa; this models history-dependent connectivity, associated with the learned sequence of stimulus 1 (activating MC1) arriving before stimulus 2 (activating MC2). The activation of MC1 helps to prime the activity of MC2. The level of excitation of the superficial layer regular spiking neurons (RS cells) is set so that if the 2 columns were not connected at all, then MC1 would oscillate at beta1, as before, but MC2 would initially be silent. However, due to the potentiation of synaptic excitation from MC1 to MC2, the IB and FS cells of MC2 are initially active (Fig. 3 *A* and *B*). From these baseline conditions, an increase in the tonic excitation of the RS cells of MC2 resulted in this whole MC becoming active, joining in the beta1 oscillation with small phase difference with MC2 (Fig. 3 *A* and *C*). Consequently, the combined beta1 power of the superficial layers of the 2 MCs greatly increased, while the beta1 power of the deep layers decreased (Fig. 3*B*). We note that in contrast to Fig. 2*G*, where the top-down input was rhythmic and was not enough to activate an MC, here it is the tonic current of the cells that is increased, presumably an effect of new sensory stimulus. The priming of MC2 by MC1, a result of prior plastic changes, is not necessary for the activation of MC2 but for the coordination of the 2 MCs. We also note that the model describes only activation of previously learned sequences and that we do not model the learning of new sequences.

### Cell Assemblies, Active at beta1 Frequency, Can Be Manipulated by Subtraction.

**Fig. 4.**
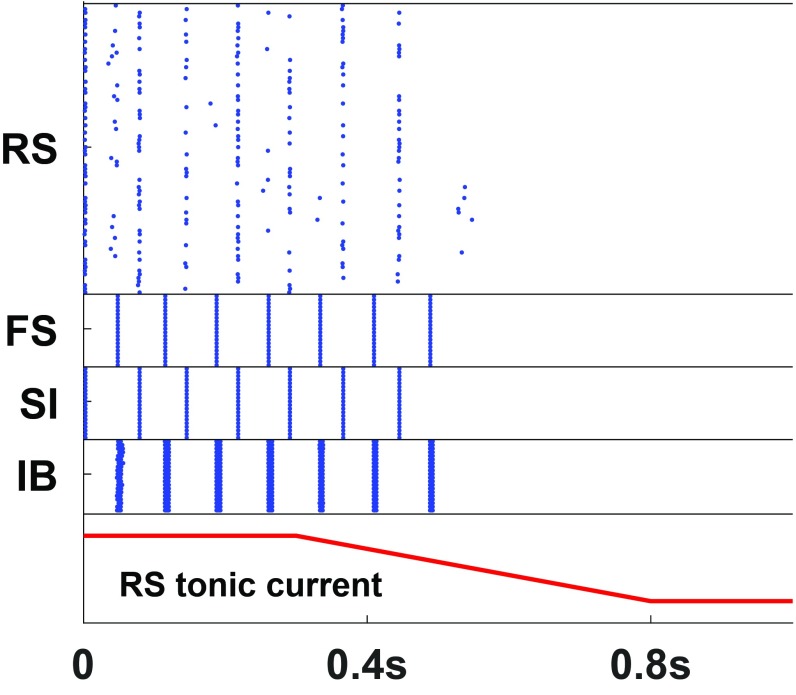
Starting at 300 ms, the tonic input to the RS cells is gradually reduced (*Materials and Methods*). The network turns off after about 200 ms. The result was consistent over 10 simulations.

**Fig. 5.**
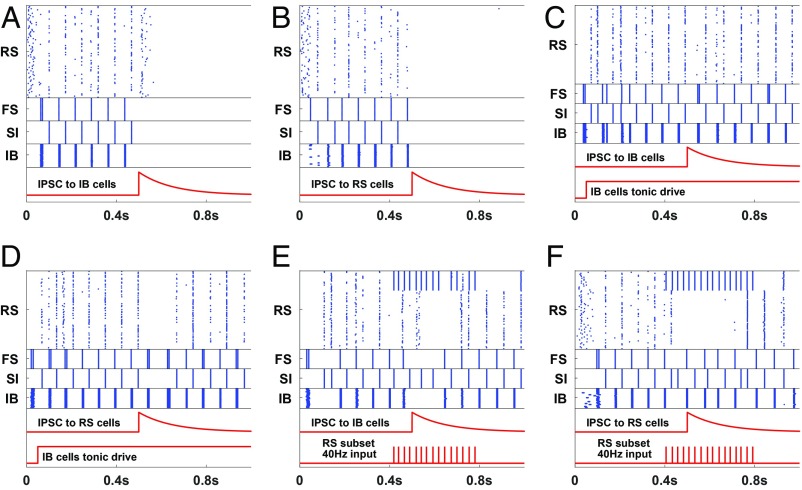
Turning off the network. (*A* and *B*) A pulse of inhibition either to the IB cells (*A*) or to the RS cells (*B*) is enough to turn off the network. The inhibitory pulse used here is in the form of synaptic inhibition, starting at 500 ms, with decay constant 120 ms (red trace). (*C* and *D*) Increased tonic drive to IB cells, presumably a result of top-down control, can prevent the network from turning off by a pulse of inhibition to the IB (*C*) or RS (*D*) cells. (*E* and *F*) Ongoing stimulation in the form of a gamma (40 Hz; bottom red trace) rhythm delivered to a subset of the RS cells prevents the network from turning off by a pulse of inhibition to the IB (*E*) or RS (*F*) cells. In all 6 cases the results were consistent over 10 simulations.

The WM buffer is considered to have a finite capacity determined by a broad array of internal and external (sensory) factors (36). Using the beta1 frequency model of the poststimulus, parietal episodic buffer, we examine 2 of these factors: 1) passive extinction and 2) effect of distractors. For passive extinction we consider the dynamic nature of the beta1 rhythm. Both IB and RS principal neurons must be sufficiently excitable to respond to each other’s inputs and maintain the iterative excitatory interaction needed for concatenation. Reducing tonic, neuromodulatory excitation to RS neurons effectively extinguished the beta1 rhythm (Fig. 4). This occurred without changes to IB neuronal excitability. To examine the effects of distractors we considered the competition for cortical space framework proposed by Adesnik and Scanziani (37). Here the influence of activity in MCs not forming part of the episodic buffer assembly generates inhibitory synaptic events in either RS or IB neurons depending on cortical origin. We did not explicitly model the inhibitory synapses; we simulated such events as inhibitory pulses of current into these cells. Fig. 5 *A* and *B* show that an inhibitory synaptic event in either layer can terminate that MC’s beta1 rhythm. However, this effect was not absolute: The terminating effects of these distractors could be effectively countered by 2 mechanisms. First, tonic neuromodulatory excitation—a key attentional mechanism (38) selectively to layer 5 neurons (39)—abolished the terminating effects of inhibitory synaptic input (Fig. 5 *C* and *D*). Second, bottom-up sensory input to RS neurons, presented concurrently with the inhibitory synaptic input, also prevented termination of that MC’s ongoing beta1 rhythm (Fig. 5 *E* and *F*).

The same mechanism can also be used to clear the buffer. The contents of WM can become irrelevant once a task is completed. The central executive must have a way of clearing the episodic buffer to allow for taking up different tasks. As already shown in Fig. 5 *A* and *B*, a pulse of inhibition is enough to turn off a beta1 MC. If this pulse is a generalized one, instead of affecting specific MCs, the whole content of the WM can be erased. A generalized inhibition could be the result, for example, of activation of neurogliaform cells, which can provide a blanket of inhibition in the deep layers (40). Recall that such inhibition cannot turn off MCs that are in use (Fig. 5 *E* and *F*).

### Top-Down Disinhibition Modulates Readout of Memory Content.

**Fig. 6.**
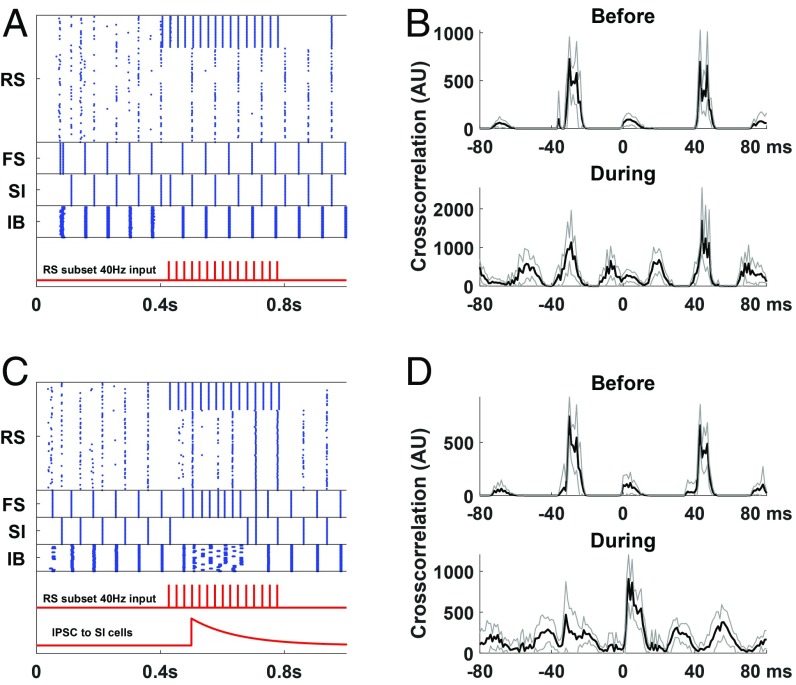
(*A*) Rastergram of a simulation of the network with 40 Hz input (red trace) to 1/4 of the RS cells (and all of the FS cells but weaker). (*B*) Cross-correlogram of RS and IB spikes before (200 to 400 ms) and during the input presentation (400 to 800 ms) for the simulation in *A*. The IB cells fire about 40 ms after the RS cells, even when the RS cells are entrained to the gamma rhythm. Note that in the “during” condition, there are bumps about every 25 ms, due to the quasi-periodicity of the input (*Materials and Methods*). (*C*) Same as *A* but with an inhibitory pulse given to the SI cells (lower red trace). (*D*) Cross-correlogram of RS and IB spikes for the simulation in *C* before the input presentation (200 to 400 ms) and during the first 150 ms of the SI inhibition (500 to 650 ms). During the input presentation the IB cells now fire shortly after the RS cells. Black lines in *B* and *D* are average of 10 simulations. Gray lines are ±1SD.

The main purpose of WM is to hold in memory sensory inputs, accumulated and manipulated over time, to guide behavioral responses to subsequent sensory inputs. Thus, a readout of the contents of the episodic buffer must differ somehow from the rehearsal inherent in the ongoing beta1 activity. Recognition of a sensory event in classical WM tasks involves the central executive (e.g., see ref. 41). In addition to the direct excitatory effects of top-down input onto principal cells, it is also recognized that somatostatin-containing interneurons (SI cells as modeled here) can be selectively silenced via activation of VIP-containing interneurons (42). We investigated this input to the episodic buffer model by comparing the effects of bottom-up sensory input, with and without a concurrent silencing of SI neurons, on the beta1 rhythm (Fig. 6). With the beta1 network intact, presentation of a gamma frequency input failed to disrupt the beta1 rhythm (Fig. 6 *A* and *B* and *SI Appendix*, Fig. S2). Instead, additional correlation peaks were seen corresponding to the input frequency (Fig. 6*B*). In contrast, concurrent silencing of SI interneurons during gamma-frequency input produced a dramatic change in the temporal organization of deep and superficial layers (Fig. 6 *C* and *D*). The resulting synchronization of these 2 cortical layers represents a collapse of the concatenation sequence during stimulus presentation. Establishing synchrony of both deep and superficial cortical layer outputs has powerful consequences for downstream targets (*Discussion*).

## Discussion

Results from the present simulations predict that the dynamic landscape underlying parietal beta1 rhythms provides a substrate for an episodic buffer component of the distributed WM model proposed by Baddeley (5). The iterative interaction between deep and superficial cortical layers permits formation of an engram on the basis of prior excitation (sensory input), updatability in the form of addition and subtraction of cortical MCs, distractability by unrelated sensory input, and a readout mechanism subsequent to representation of the original input(s). All of these properties were shown to be under the control of top-down inputs from a model central executive.

A number of elegant models of WM have focused on the prefrontal cortex—the presumed central executive. Building on the seminal work of Goldman-Rakic (43), persistent activity, following a transient excitation in layer 2/3 prefrontal principal cells, has been used to represent the engram (29). Expansion of these computational models to include persistent activity in parietal cortex predicted complex behaviors that may account for some of the core features of WM (44). The present model differs from this approach in 2 main ways.

First, the parietal component in ref. 44 does not involve persistence of the type characterized in prefrontal cells. The persistence in the above models is generated at a single neuron level by a combination of intrinsic conductances (45) under the control of attention-related neuromodulators (46). Population-level expression and control is predicted to be mediated by the level of recurrent NMDA receptor-mediated excitation and local circuit inhibition (29). In contrast, the persistent activity we focus on is an emergent property of local neuronal connections distributed across deep and superficial layers—the beta1 frequency rhythm (22, 23). Multiple neuronal subtypes in multiple cortical laminae have to combine synergistically to produce beta1.

Second, the distribution of local network components required for the beta1 rhythm across different laminae allows for a much richer pattern of interaction with top-down (central executive) inputs. Prefrontal projections to lower hierarchies of neocortex are extremely complex. Regional projections are highly parcellated (47, 48) and very diverse in terms of target laminae (49). We took advantage of this complexity to model the core features of WM discussed below.

A similar model was used by Kopell et al. (28) to study the interaction of novel and familiar stimuli. The cell types used there were the same as in our model (we have changed the nomenclature of the LTS cells to slow inhibitory [SI], to accurately reflect what the cells are doing in the simulations), and the connections were similar. Novel and familiar stimuli, modeled as different levels of tonic input (to account for habituation), were presented to different subsets of the RS cells. The authors showed that the 2 stimuli can both be represented in the same column, without competing with each other. Our model differed in a number of ways, consistent with the different focus of the current work, which was to examine the suitability of the parietal beta1 rhythm as a substrate of the episodic buffer. First, although the basal network we considered was similar to what constituted a cortical column in ref. 23, we used it to represent a minicolumn. That is, we considered each of these MCs as the smallest functional unit and did not allow for multiple items to be stored in the same MC. Instead, we looked explicitly at the interactions between different MCs. Second, we also investigated the effect of top-down control, in particular its ability to coordinate multiple MCs. Third, we also looked at rhythmic input, rather than just tonic input, consistent with evidence that both sensory signals and executive control inputs can come in the form of rhythmic activity (50). Finally, we modeled additional scenarios that are relevant to WM function, such as removing an item from the buffer or modulating the readout.

Other models of WM make use of oscillations (51–53). The model closest in spirit to ours is the work by Dipoppa and Gutkin (51). That model is set in prefrontal cortex and uses integrate-and-fire neurons. There, background input at different frequencies allows the storage and/or release of memories. Our current model differs from that in multiple important ways. First, it is set in parietal cortex, making use of detailed, biophysical properties of that cortex. More crucially, it stresses the working aspect of WM; the cell assemblies that are created in our model not only are stored but are also capable of being manipulated. This manipulation was only possible through the mechanistic flexibility afforded by the diverse complement of biophysical properties underlying the network behavior described here.

To set up an episodic buffer for use in WM, our model showed that parietal cortical MCs generating local beta1 activity could be bound via synchronization by a range of patterned top-down excitatory inputs to layer 5 known to be generated by prefrontal cortex [beta–gamma frequencies (54)] (Fig. 2). Thus, the model is consistent with the critical dependence on frontal–parietal interactions for manifesting WM (55). The induced synchronization in the model lasted for several hundred milliseconds after the termination of the brief top-down input. Notably, in the case of gamma input (40 and 80 Hz) all of the MCs continued firing in phase, increasing the total beta1 power (Fig. 2 *C*, *E*, and *F*), while in the case of beta1 (15 Hz) or beta2 (25 Hz) input, after the input termination, groups of MCs were often firing antiphase, increasing the total power in the frequency twice that of the individual MCs, which happened to be in the beta2 range (Fig. 2 *B*, *E*, and *F*). Gamma and beta2 are often seen in PFC WM (30–32).

Frontal–parietal interactions also underlie the core property of WM that makes it distinct from other memory subtypes: It is updatable. The content held in WM is labile, being continuously selectable by processes of subtraction and addition following the initial selection of components, again via top-down inputs from prefrontal cortex (56–58). The simulations here demonstrate that beta1 activity can simply fade away over time (Fig. 4) or be actively terminated by a top-down signal but not in MCs that are in use (Fig. 5).

In our model, adding new content simply corresponds to activating new cortical MCs. As discussed earlier, Kopell et al. (28) have shown that novel and familiar stimuli can be represented in a single beta1 column without competing with each other. Here we focused on the possibility of unifying the representation of a stimulus that arrives at a previously inactive MC with items represented in different MCs. If the new MC activated is associated with an already active MC, for example, due to previous learning, our simulations predict that these MCs will coordinate, resulting in a large increase of the total beta1 power in the superficial layers and a smaller decrease in the deep layers (Fig. 3). The increase in beta1 power is consistent with behavioral experiments regarding speech processing, where presentation of a syntactically correct sentence is associated with increase in beta1 activity over time (59). Although we do not explicitly model the learning of novel sequences, the connectivity we use for already learned sequences is consistent with spike time-dependent plasticity: the connections go from cells encoding a previous stimulus to ones encoding the later one.

The behavioral performance of WM is exquisitely sensitive to distractors—sensory inputs unrelated to the information held in WM. We modeled this implicitly as inhibition thought of as coming from corticocortical connections (37) and showed that distractors, in this mechanistic form, can be disruptive to the beta1 activity. However, this effect could be overcome either by strong top-down (central executive) excitatory input to the MC included in WM (e.g., ref. 60) or by the concurrent representation of the MC-specific bottom-up excitatory input (Fig. 5).

Synaptic inhibition was also predicted to be vital for readout from the episodic buffer. In prefrontal cortex (i.e., central executive regions), reduced parvalbumin- or somatostatin-containing interneuron function has been shown to be detrimental to short-term memory function, primarily through false outputs in a go–no go task (41). In the same study, enhancing VIP-containing interneuron function improved memory performance. Our simulations suggest a similar dependence on inhibition in parietal cortex. Slow inhibition, modeled as from somatostatin-containing interneurons, is a vital feature of beta1 rhythms (23), and top-down prefrontal cortical inputs to lower hierarchical regions activate VIP-containing interneurons, thus selectively inhibiting these somatostatin-containing cells (61). Representation of an MC’s bottom-up input preserves the beta1 activity but adds additional superficial layer principal cell activity within each beta1 period (Fig. 6 *A* and *B*). This can only occur if slow synaptic inhibition is functional. If it is selectively removed by an inhibitory synaptic event onto the source interneurons, there is an overt phase change between superficial and deep layer principal cell spiking (Fig. 6 *C* and *D*). This enhanced synchrony of deep and superficial output neurons in the WM MC can result in a synergistic effect that would increase their impact on target regions, which can be interpreted as some aspect of readout of memory content. That could also possibly affect short-term synaptic plasticity (62). Note that a continuous readout from the superficial and deep layers can be ongoing to various other parts of the brain participating in WM, allowing further manipulation of the content. Our point here is that the disinhibition allows the synchronization of superficial and deep layers at a particular time, so that their synergistic effect may elicit a further desired outcome.

Although the physiology of the parietal rhythm used in the model was motivated by mechanistic observations from an in vitro preparation, there are multiple examples in human and nonhuman primates in vivo in which this beta1 rhythm has been noted in parietal cortex and in which the task involved would benefit from the functional properties described above. Already mentioned is the work of Bastiaansen et al. (59) on buildup of beta1 during syntactically correct speech. A similar paper about involvement of buildup of beta during perceptual decision making is ref. 63. Arnal et al. (64) found an increase in beta1 phase-locking in the parietal cortex when there was a violation of audiovisual expectation, with a strong gamma input to PC likely producing the beta1 rhythm. We also note that both high and low beta activity accompanies burst activity in prefrontal cortex and that burst firing increases specifically following attention cues, suggesting that the sensory feature is successfully uploaded in the PFC (65). Since there is strong interaction between the PC and the PFC, the beta1 rhythm in the former may be important in the creation of the beta burst in the PFC. We note that although we show ongoing beta1 in our simulations, in vivo, such dynamics may be short lived, i.e., a few cycles of beta.

Our results make predictions for lamina-specific spike phase relationships and changes in levels of neural activity during different conditions of WM function. For example, the readout mechanism suggests that when the contents of WM are being released, there should be a clear shift in the phase relationship between deep and superficial pyramidal cells, from out of phase to near synchrony. This can be tested in a WM behavioral experiment where deep and superficial layers are recorded simultaneously. The results of Fig. 5 suggest that a distractor presented concurrently with the input is less likely to lead to erasure of the memory. Also, the results of Fig. 2 suggest that during binding of features, a brief top-down input (<150 ms) of beta1 or higher frequency is enough to trigger synchronization in the parietal cortex (increase of power in beta1 and/or beta2 bands) that is sustained for hundreds of milliseconds. Finally, Fig. 3 refers to a condition where a temporal association between 2 items has already been established. If the MCs associated with the 2 items have been identified, then the prediction is that after presentation of item 1 and before presentation of item 2, deep layer pyramidal cells (IB cells) of MC2 will be active and synchronized with those of MC1, but superficial pyramidal cells (RS cells) of MC1 will be inactive. After presentation of item 2, RS cells of MC1 will also be active and synchronized with those of MC1.

We note that the above predictions are all qualitative, rather than quantitative; we did not look into the exact parameter ranges for which the observed phenomena hold. Given the simplified nature of the model, such quantitative statements would be of limited value.

In summary, the parietal cortex is an ideal locus for the episodic buffer component of WM given its convergent connections from multiple sensory slave systems and parcellated connections with frontal, central executive regions. The complexity predicted to be required for the functional episodic buffer (7) appears to be inherent in the beta1 rhythm as manifest in this region. Our simulations reproduce many of the core features of WM and demonstrate that it is the interaction between deep and superficial cortical layers, and their respective corticocortical inputs, which provides the balance of robustness and manipulability that defines WM. However, the dependence of beta1 rhythms on intrinsically bursting (IB) neurons suggests involvement of subcortical structures too. This cell type specifically sends outputs to subcortical structures (66, 67). Many of these targets are involved in WM (68) as evidenced by the selective effects of subcortical stroke on WM (69). Further studies are therefore required to expose the role of such structures on mechanisms of WM.

## Materials and Methods

We used a modified version of the model of a parietal cortex (area S2) column from ref. 23 (Fig. 1*A*). The model involves only superficial (L2/3) and deep (L5) layers. There are 3 cell types in the superficial layers, regular spiking (RS), fast spiking (FS), and slow inhibitory (SI) neurons, all modeled as single compartments. In the deep layers there is only 1 cell type, intrinsically bursting cells (IB), which in S2 are known to be able to produce a beta2 on their own (70). IB cells are modeled as consisting of 4 compartments: apical dendrite, basal dendrite, soma, and axon. The RS and IB cells are excitatory, while the FS and SI cells are inhibitory. Each cell/compartment is modeled as a Hodgkin–Huxley neuron possibly with extra currents, including h current, M current, and a high-threshold calcium current.

The connectivity with chemical synapses is as shown in Fig. 1*A* and described in detail in *SI Appendix*. There are gap junctions between all pairs of SI cells and all pairs of IB axons. The electrical continuity of the different compartments of each IB cell is also modeled with gap junctions.

Input was modeled as current through synaptic conductances that responded to externally controlled electric potentials. For periodic input, the length of each period varied randomly around the nominal value.

See *SI Appendix* for details of the model.

### Statistics.

All analyses were performed in MATLAB (The MathWorks, Inc.). Spike trains were calculated and summed over groups of cells as described in each figure. Cross-correlograms were computed for the summed spike trains, and power spectra were then computed as the discrete Fourier transform of the cross-correlograms. Beta1 power was calculated as the peak in the power spectrum in the band 12 to 20 Hz and compared across conditions using the Wilcoxon rank-sum test.

## Supplementary Material

Supplementary File
