# Supplementary material for: Parietal low beta rhythm provides a dynamical substrate for a working memory buffer
Source: Proc Natl Acad Sci U S A. 2019 Aug 1;116(33):16613–20. doi: 10.1073/pnas.1902305116 (PMC6697799; doi:10.1073/pnas.1902305116)
Supplement: Supplementary File [file pnas.1902305116.sapp.pdf]

PNAS
www.pnas.org

Supplementary Information for

Parietal low beta rhythm provides a dynamical substrate for a working memory buffer.

Alexandros Gelastopoulos, Miles A. Whittington, and Nancy J. Kopell

Nancy J. Kopell.
E-mail: nk@bu.edu

This PDF file includes:

Supplementary text
Figs. S1 to S2
Tables S1 to S6
References for SI reference citations

www.pnas.org/cgi/doi/10.1073/pnas.1902305116

Supporting Information Text

Effect of top-down input duration

In Fig. 2 of the main text we showed that a common top-down input of duration 150 ms and sufficientl high frequency (15 Hz or higher) to a number of weakly interconnected columns resulted in the columns synchronizing with each other, and this effect lasted even after the input went away. Fig. S1 shows that the columns synchronize even if the input duration is made shorter, but not if it is reduced to a single pulse.

Gamma input of different frequencies to a single beta1 column.

In Fig. 6a,b of the main text we showed that a 40 Hz input presented to the superficial layers alone was not enough to disrupt the beta1 rhythm of a parietal column. Moreover, the IB cells kept firing with high probability about 40 ms after the RS cells. Fig. S2 shows that this is true even for higher frequency input, but for 90 Hz input IB cells also often fire immediately after RS cells.

Supplementary Methods

In this section we give a detailed description of the model used for the simulations.

The network. We use a modified version of the model of a parietal cortex (S2) column from (1). The model involves only superficial (L2/3) and deep (L5) layers. There are three cell types in the superficial layers, Regular Spiking (RS), Fast Spiking (FS) and Slow Inhibitory (SI) neurons (LTS neurons in (1)), all modeled as single compartments. In the deep layers there is only one cell type, Intrinsically Bursting cells (IB), modeled as consisting of four compartments: apical dendrite, basal dendrite, soma, and axon. The RS and IB cells are excitatory, while the FS and SI cells are inhibitory. The details of the cell models are described below and they are the same as in (1), unless otherwise specified.

In one cortical column we include 80 RS cells and 20 cells of each other type. The connectivity with chemical synapses is described in Table S3. There are gap junctions between all pairs of SI cells and all pairs of IB axons (Table S4). The electrical continuity of the different compartments of each IB cell is also modeled with gap junctions (Table S4).

In the simulations of Fig. 3 we include two columns with synapses from IB cells of column 1 to IB cells of column 2 (Table S3). In the simulations of Fig. 2 we include eight columns, each with 20 RS cells and 5 cells of each other type, with synapses between RS cells and between IB cells of different columns (Table S3).

Model equations. Each cell/compartment is modeled as a set of ordinary differential equations, describing the membrane potential and the ionic current gating variables. The membrane potential of a cell/compartment evolves according to

$$\begin{aligned}
C\frac{d\mathbf{V}}{dt} = &- J - I_{syn} - I_{gap} - I_{ran} - I_{app} \\
&- g_L \cdot (\mathbf{V} - V_L) \\
&- g_{Na} \cdot m_0^3(\mathbf{V}) \cdot \mathbf{h} \cdot (\mathbf{V} - V_{Na}) \\
&- g_K \cdot \mathbf{m}^4 \cdot (\mathbf{V} - V_K) \\
&- g_{AR} \cdot \mathbf{m_{AR}} \cdot (\mathbf{V} - V_{AR}) \\
&- g_{KM} \cdot \mathbf{m_{KM}} \cdot (\mathbf{V} - V_{KM}) \\
&- g_{CaH} \cdot \mathbf{m_{CaH}^2} \cdot (\mathbf{V} - V_{CaH}).
\end{aligned}$$

where: C is the capacitance, J is a tonic current, I_{syn} is the total synaptic current, I_{gap} is the total current through gap-junctions, I_{ran} is additive noise, I_{inp} is an applied current (input), g_x denotes the conductance of current x, where $x = \{L, Na, K, AR, KM, CaH\}$, V_x denotes the reversal potential for current x, and m, m_0, h and $\mathbf{m_x}$ denote gating variables. Variables in boldface denote state variables. The subscripts stand for the following: L - leak current, Na - fast inactivating sodium current, K - delayed rectifier potassium current, AR - h-current, KM - M-current, CaH - high-threshold calcium current. The h-current is present only in the RS and SI cells, and in the IB dendrites. The M-current is present only in IB dendrites and axons. The high-threshold calcium current is present only in IB dendrites. All other ionic currents are present in all cells/compartments. The currents I_{syn}, I_{gap}, I_{ran} and I_{inp} are described below. The values of all other parameters and the function $m_0(\mathbf{V})$ for each of the cells/compartments are given in Tables S1 and S2. In these and in following Tables, conductance, capacitance, and electric currents are given per unit area of the cell surface, since the actual size of a cell is irrelevant. All values are given in the following units: conductance - mS/cm^2, capacitance - $\mu F/cm^2$, electric current - $\mu A/cm^2$, electric potential - mV, time - ms.

The rest of the state variables follow first-order dynamics, with membrane potential-dependent dynamics. In accordance with (1), we describe the time evolution of $\mathbf{h}, \mathbf{m}$, and $\mathbf{m_{AR}}$ in terms of the steady-state values and time constants, and that of $\mathbf{m_{KM}}$ and $\mathbf{m_{CaH}}$ in terms of the forward and backward rates:

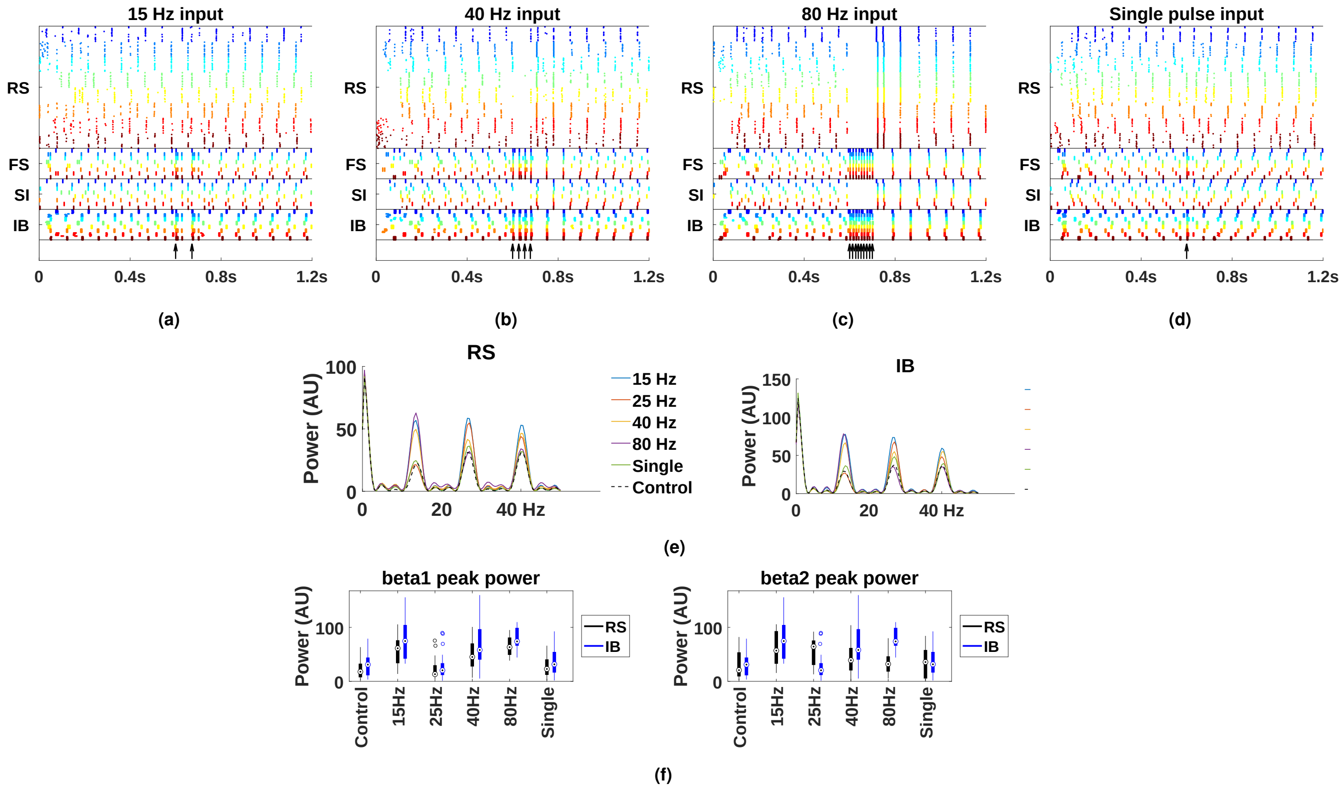

Fig. S1. Rhythmic input to the deep layers is less efficient in synchronizing beta1 columns, if its duration is too short. **(a-c)** As in Fig. 2 of the main text, the same input is given to the deep layers of each of 8 beta1 columns, of frequency (a) 15 Hz, (b) 40 Hz, or (c) 80 Hz, but now lasting from 600-700 ms (blue arrows). **(d)** As in (a-c), but for a non-rhythmic, single-pulse input. **(e)** Power spectrogram after the input presentation (900-1200 ms). Beta1 power peak is significantly higher compared to control when the input frequency is 15 ($p_{RS} = 5.8 \cdot 10^{-5}$, $p_{IB} = 7.22 \cdot 10^{-0.5}$), 40 ($p_{RS} = 0.0011$, $p_{IB} = 0.0015$), or 80 Hz ($p_{RS} = 6 \cdot 10^{-7}$, $p_{IB} = 1.34 \cdot 10^{-6}$), but not when the input frequency is 25 Hz ($p_{RS} = 0.54$, $p_{IB} = 0.71$) or in the case of a single pulse ($p_{RS} = 0.32$, $p_{IB} = 0.16$). Beta2 power peak is significantly higher compared to control when the input frequency is 15 ($p_{RS} = 0.0017$, $p_{IB} = 0.0018$) or 25 Hz ($p_{RS} = 0.003$, $p_{IB} = 0.0028$), but not when input frequency is 40 ($p_{RS} = 0.1$, $p_{IB} = 0.057$) or 80 Hz ($p_{RS} = 0.29$, $p_{IB} = 0.39$), or in the single input pulse case ($p_{RS} = 0.33$, $p_{IB} = 0.15$).

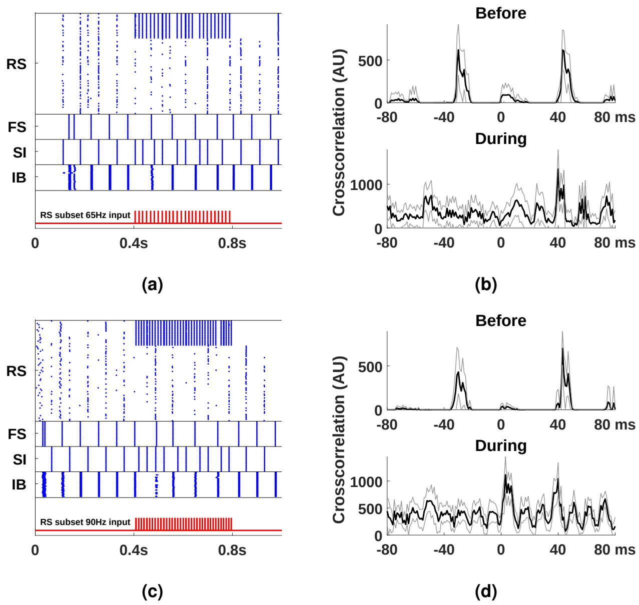

Fig. S2. (a) Rastergram of a simulation of the network with 65 Hz input (red trace) to 1/4 of the RS cells (and all of the FS cells, but weaker). **(b)** Crosscorrelogram of RS and IB spikes before (200-400 ms) and during the input presentation (400-800 ms) for the simulation in (a). As with 40Hz input (Fig. 6a,b), the IB cells fire about 40 ms after the RS cells, even when the RS cells are entrained to the gamma rhythm. (c,d) Same as (a,b), but for 90 Hz input. Now the IB cells also often fire shortly after the RS cells. Black lines in (b,d): average of 10 simulations; gray lines: ± 1 s.d. AU: arbitrary units.

$$\frac{d\mathbf{x}}{dt} = \frac{1}{\tau_x(\mathbf{V})} \cdot (x_\infty(\mathbf{V}) - \mathbf{x}), \qquad x = \{m, h, m_{AR}\}$$

$$\frac{d\mathbf{x}}{dt} = \alpha_x(\mathbf{V}) \cdot (1 - \mathbf{x}) - \beta_x(\mathbf{V}) \cdot \mathbf{x}, \qquad x = \{m_{KM}, m_{CaH}\}$$

The expressions for the functions $\tau_x(\mathbf{V})$, $x_\infty(\mathbf{V})$, $\alpha_x(\mathbf{V})$, and $\beta_x(\mathbf{V})$ are given in Table S2. Note that the two descriptions are equivalent, and the relation between the (τ_x, x_∞) and the (α_x, β_x) descriptions is given by the equations:

$$\tau_x = \frac{1}{\alpha_x + \beta_x}, \qquad x_\infty = \frac{\alpha_x}{\alpha_x + \beta_x} \qquad \alpha_x = \frac{x_\infty}{\tau_x} \qquad \beta_x = \frac{1 - x_\infty}{\tau_x}.$$

As in (1), we make the following adjustments for particular cells/compartments, relative to the values in Table S2: for the IB axons, we multiply the forward rate α_{KM} of $\mathbf{m_{KM}}$ by 1.5 and its backward rate β_{KM} by 1.25. For the IB apical and basal dendrites, we multiply both the forward and backward rates of $\mathbf{m_{CaH}}$ by 3, the forward rate of $\mathbf{m_{AR}}$ by 2.75, and the backward rate of $\mathbf{m_{AR}}$ by 3. For the RS cells, we multiply the forward rate of $\mathbf{m_{AR}}$ by 3.5 (1.75 in (1)) and leave the backward rate unchanged (multiplied by 0.5 in (1)).

The synaptic current I_{syn} is the sum of the synaptic currents $I_{syn,i}$, one for each incoming synapse for the given cell/compartment. The contribution of each synapse is given by

$$I_{syn,i} = \mathbf{s_i} \cdot g_i \cdot (\mathbf{V} - V_i), \qquad [1]$$

where $\mathbf{s_i}$ is the synapse gating variable, g_i is the maximal conductance, $\mathbf{V}$ is the membrane potential of the post-synaptic cell, and V_i is the reversal potential. The synaptic gating variable $\mathbf{s_i}$ evolves according to the equation

$$\frac{d\mathbf{s_i}}{dt} = -\frac{\mathbf{s_i}}{\tau_{d,i}} + \frac{1 - \mathbf{s_i}}{\tau_{r,i}} \cdot 0.5 \left(1 + \tanh \frac{\mathbf{V_{pre,i}}}{10}\right), \qquad [2]$$

where $\tau_{d,i}$ and $\tau_{r,i}$ are the decay and rise time constants, respectively, $\mathbf{V_{pre,i}}$ is the membrane potential of the pre-synaptic cell, and tanh denotes the hyperbolic tangent function. The maximal conductances, reversal potentials, decay constants, and rise constants for the various synapses are given in Table S3.

The gap-junctional current I_{gap} is the sum of the gap-junctional currents $I_{gap,i}$, one for each gap-junction involving the given cell/compartment. The contribution of each gap-junction is given by

$$I_{gap,i} = g_i \cdot (\mathbf{V} - \mathbf{V_i'}),$$

where g_i is the conductance, $\mathbf{V}$ is the membrane potential of the cell/compartment in question, and $\mathbf{V_i'}$ the membrane potential of the other cell/compartment that the gap-junction involves. The conductances for the various gap junctions are given in Table S4.

The term I_{ran} describes a stochastic input to the cells and it is the sum of a white noise term and an excitatory input modeled as synaptic current, activated stochastically according to a Poisson process and decaying exponentially. That is,

$$I_{ran} = \sigma_{ran}^2 \cdot dW + \mathbf{s_{ran}} \cdot g_{ran} \cdot (\mathbf{V} - V_{rev,ran}),$$

where dW is Gaussian white noise of unit variance per ms, σ_{ran}^2 is the intensity of this additive white noise, g_{ran} is the Poisson synaptic input conductance, $V_{rev,ran}$ its reversal potential, and $\mathbf{s_{ran}}$ evolves according to

$$\frac{d\mathbf{s_{ran}}}{dt} = -\frac{1}{\tau_{ran}} \cdot \mathbf{s_{ran}} + \sum_i \delta(t - t_i),$$

where $\delta(t - t_i)$ denotes the delta function centered at t_i, and the times $\{t_i\}$ are a realization of a Poisson process with rate λ. The values of the parameters used are given in Table S1.

The term I_{inp} is a sum of currents $I_{inp,i}$ also following Eq. 1 and 2, but in this case instead of the presynaptic membrane potential $\mathbf{V_{pre,i}}$, we have an externally controlled potential $\mathbf{V_{inp,i}}$, which represents a single pulse or a train of pulses. At each pulse, $\mathbf{V_{inp,i}}$ is instantaneously set to a fixed value V_{high} and then decays exponentially to V_{low}. That is,

$$\begin{aligned} I_{inp,i} &= \mathbf{s_{inp,i}} \cdot g_{inp,i} \cdot (\mathbf{V} - V_{rev,inp,i}), \\ \frac{d\mathbf{s_{inp,i}}}{dt} &= -\frac{\mathbf{s_{inp,i}}}{\tau_{d,inp,i}} + \frac{1 - \mathbf{s_{inp,i}}}{\tau_{r,inp,i}} \cdot 0.5 \left(1 + \tanh \frac{\mathbf{V_{inp,i}}}{10}\right), \\ \frac{d\mathbf{V_{inp,i}}}{dt} &= \frac{1}{\tau_{inp}} (V_{low} - \mathbf{V_{inp,i}}) + (V_{high} - \mathbf{V_{inp,i}}) \cdot \sum_j \delta(t - t_{i,j}), \end{aligned} \qquad [3]$$

where $t_{i,j}$ are the times of the pulses. When the input is periodic with nominal frequency f, the time $t_{i,j+1} - t_{i,j}$ between two pulses is a random number, uniformly distributed in the interval $\left(\frac{0.9}{f}, \frac{1.1}{f}\right)$, independently chosen for each i, j. Details for the various inputs are given in Table S5. For the tonic inputs in Fig. 2, 4, 5, and 6 we adjust the value of J (see caption of Table S1).

Initial conditions. To randomize initial conditions, all state variables are initialized at values chosen uniformly randomly from an interval (Table S6). Moreover, at the beginning of the simulation an inhibitory pulse is given to RS cells, following the dynamics of equation 3, identical for RS cells of the same column, but of different magnitude for different columns, chosen randomly and independently (Table S5). Similarly, an initial excitatory pulse is given to the IB cells, following the dynamics of equation 3, same for IB cells of the same column, but at different times for different columns, with the starting time independently uniformly randomly chosen from an interval (Table S5). An exception are the last four columns in Fig. 1e and 2g whose IB cells do not receive such pulse. All synapse gating variables $\mathbf{s_i}$ and input gating variables $\mathbf{s_{inp,i}}$, as well as $\mathbf{s_{ran}}$, are initialized at 0.

References

1. Mark A Kramer, Anita K Roopun, Lucy M Carracedo, Roger D Traub, Miles A Whittington, and Nancy J Kopell. Rhythm generation through period concatenation in rat somatosensory cortex. *PLoS computational biology*, 4(9):e1000169, 2008.

Table S1. Parameters for the various cells/compartments. All values are taken from (1), except for the parameter J (all cells) and g_{AR} for RS cells and IB apical dendrites. Parameters independent of the cell/compartment: $C = 0.9$, $V_{rev,ran} = 0$, $\tau_{ran} = 4$, $\lambda_{ran} = 0.1/ms$. *Exceptions for J: In Fig. 5c,d, we use the following values for the IB compartments: apical dendrite 25.5, basal dendrite 42.5, soma -4.5, axon -0.4. In Fig. 4, we start from normal values, but between 300-800 ms we reduce the value for RS at a rate 0.05/ms. In Fig. 3, we use the value 65 for the RS cells of column 2 and, starting at some randomly chosen time in the interval $[500, 570]$, we let it decay exponentially towards 25, with decay constant 50 ms. In the tonic input condition of Fig. 2, between 600-750 ms we use the value 33.5 for the IB basal dendrites. **Exceptions for g_{ran}: In Fig. 1e and 2g we use the value $g_{ran} = 0.17$ for the RS cells of the last four columns.

var	RS	FS	SI	IB apical dend.	IB basal dend.	IB soma	IB axon
J^*	25	35	50	27.5	44.5	-3.5	0.1
g_L	1	1	6	2	2	1	0.25
g_{Na}	200	200	200	125	125	50	100
g_K	20	20	10	10	10	10	5
g_{AR}	40	-	50	180	115	0	0
g_{KM}	-	-	-	0.75	0.75	-	1.5
g_{CaH}	-	-	-	-	-	6.5	6.5
V_L	-70	-65	-65	-70	-70	-70	-70
V_{Na}	50	50	50	50	50	50	50
V_K	-95	-100	-100	-95	-95	-95	-95
V_{AR}	-35	-	-35	-25	-25	-	-
V_{KM}	-	-	-	-95	-95	-	-95
V_{CaH}	-	-	-	125	125	-	-
σ^2_{ran}	0.15	0.05	0.05	0.005	0.005	0	0.025
g^{**}_{ran}	0.03	0	0	0	0	0	0

Table S2. Expressions for functions involved in the dynamics of the ionic current gating variables, taken from (1). $\mathbf{V}$ is measured in mV. $V_0 = -87.5$ for RS cells, and $V_0 = -75$ for other types of cells/compartments.

**Variable**	τ_x	x_∞
$\mathbf{m}$ (excitatory cell)	$0.25 + 4.35 \cdot e^{-\frac{\lvert\mathbf{V}+10\rvert}{10}}$	$\left(1 + e^{\frac{-\mathbf{V}-29.5}{10}}\right)^{-1}$
$\mathbf{h}$ (excitatory cell)	$0.15 + \frac{1.15}{1+e^{\frac{\mathbf{V}+33.5}{15}}}$	$\left(1 + e^{\frac{\mathbf{V}+59.4}{10.7}}\right)^{-1}$
$\mathbf{m}$ (inhibitory cell)	$0.25 + 4.35 \cdot e^{-\frac{\lvert\mathbf{V}+10\rvert}{10}}$	$\left(1 + e^{\frac{-\mathbf{V}-27}{11.5}}\right)^{-1}$
$\mathbf{h}$ (inhibitory cell)	$0.225 + \frac{1.125}{1+e^{\frac{\mathbf{V}+37}{15}}}$	$\left(1 + e^{\frac{\mathbf{V}+58.3}{6.7}}\right)^{-1}$
$\mathbf{m_{AR}}$	$\left(e^{-14.6-0.086\mathbf{V}} + e^{-1.87+0.07\mathbf{V}}\right)^{-1}$	$\left(1 + e^{\frac{\mathbf{V}-V_0}{5.5}}\right)^{-1}$

**Variable**	α_x	β_x
$\mathbf{m_{KM}}$	$\frac{0.02}{1+e^{\frac{-\mathbf{V}-20}{5}}}$	$0.01e^{\frac{-\mathbf{V}-43}{18}}$
$\mathbf{m_{CaH}}$	$\frac{1.6}{1+e^{-0.072(\mathbf{V}-5)}}$	$\frac{0.02(\mathbf{V}+8.9)}{e^{\frac{\mathbf{V}+8.9}{5}}-1}$

**Function**	**Expression**
$m_0(\mathbf{V})$ (excitatory cell)	$\left(1 + e^{\frac{-\mathbf{V}-34.5}{10}}\right)^{-1}$
$m_0(\mathbf{V})$ (inhibitory cell)	$\left(1 + e^{\frac{-\mathbf{V}-38}{10}}\right)^{-1}$

Table S3. Parameters for chemical synapses. The values for τ_r, τ_d and V_{rev} are taken from (1), whenever applicable (note however that values for τ_r appear to be half as large as the ones in (1), because of a difference in equation 2 from the corresponding equation in (1)). The synapses RS→RS, RS→IB (AMPA and NMDA) and SI→FS, as well as the intercolumnar synapses (last three lines) do not appear in (1). The last column indicates the number of synapses of the corresponding type for each cell in the presynaptic population. The postsynaptic cells are chosen randomly. The indication "all" means that there is a synapse for each pair of presynaptic and postsynaptic cells. For the RS→IB synapses, for each RS cell the AMPA and NMDA targets are the same. All outgoing synapses from an IB cell are from the axon compartment and all incoming synapses are to the apical dendrite, except incoming synapses from other IB cells, which are to the basal dendrite. The values for the maximal conductance g refer to a network of 80 RS cells and 20 cells of each other type; they are proportionally adjusted for different population sizes/number of synapses per cell.

Synapse	g	τ_r	τ_d	V_{rev}	# synapses
RS→RS	$\frac{1}{160}$	0.125	1	0	all
RS→FS	$\frac{1}{40}$	0.125	1	0	all
RS→SI	0.225	1.25	1	0	all
RS→IB AMPA	$\frac{1}{60}$	0.125	1	0	3
RS→IB NMDA	$\frac{1}{240}$	12.5	125	0	3
FS→RS	6.25	0.25	5	-80	all
FS→FS	2	0.25	5	-75	1 (self)
FS→SI	0.4	0.25	6	-80	all
SI→RS	0.125	0.25	20	-80	all
SI→FS	0.2	0.25	20	-80	all
SI→SI	7	0.25	20	-80	1 (self)
SI→IB	0.4	0.25	20	-80	all
IB→FS	0.2	0.125	1	0	all
IB→SI	0.045	1.25	50	0	all
IB→IB	$\frac{1}{500}$	0.25	100	0	all
RS→RS (different column - Fig. 2)	$\frac{3}{1400}$	0.125	1	0	all
IB→IB (different column - Fig. 2)	$\frac{3}{350}$	0.25	100	0	all
IB→IB (column 1 to column 2 - Fig. 3)	0.06	0.25	100	0	5

Table S4. Conductances of the various gap junctions within a column. The first six lines refer to connections within different compartments of the same IB cell and values are taken from (1). Conductance of connections in the opposite direction may differ, reflecting differences in the size of the two compartments. The last two lines refer to gap junctions between different cells, with all-to-all connectivity (within a column).

Gap junction	g
IB soma→IB apical dendrite	0.2
IB soma→IB basal dendrite	0.2
IB soma→IB axon	0.3
IB apical dendrite→IB soma	0.4
IB basal dendrite→IB soma	0.4
IB axon→IB soma	0.3
IB axon→IB axon	0.0025
SI→SI	0.2

Table S5. Parameters of the various input currents. The variable u denotes a random number, uniformly chosen from the interval $[0, 1]$.

**Input type**	g_{inp}	$V_{rev,inp}$	$\tau_{d,inp}$	$\tau_{r,inp}$	**Starting time (ms)**	**Situation modeled**
IB basal dend. rhythmic input (Fig. 2)	3	0	0.1	0.5	∼300	top-down (binding) input
RS/FS rhythmic input (Fig. 5e,f, 6)	30/0.1	0	0.1	0.5	∼400	bottom-up input
IB apical dend. single pulse (Fig. 5a,c,e)	1	-80	120	0.1	500	distractors/top-down driven termination
RS single pulse (Fig. 5b,d,f)	1	-80	120	0.1	500	distractors/top-down driven termination
SI single pulse (Fig. 6c,d)	7	-80	150	0.1	500	top-down disinhibition
RS single initial pulse (all figures)	$0.2e^{6u}$	-80	0.1	30	0	random initialization
IB soma single initial pulse (all figures except 1e and 2g)	0.15	0	0.1	150	$160u$	random initialization

Table S6. Initial values for state variables. For each cell/compartment, the initial value is chosen randomly and independently from inside an interval. The values shown here are the minimum and maximum values of this interval.

variable	RS	FS	SI	IB apical dend.	IB basal dend.	IB soma	IB axon
$\mathbf{V}$	-70/-60	-110/-100	-100/-90	-100/-90	-100/-90	-100/-90	-100/-90
$\mathbf{h}$	0/0.05	0/0.05	0/0.05	0/0.05	0/0.05	0/0.05	0/0.05
$\mathbf{m}$	0/0.05	0/0.05	0/0.05	0/0.05	0/0.05	0/0.05	0/0.05
$\mathbf{m_{AR}}$	0.035/0.06	-	0.02/0.06	0/0.001	0/0.001	-	-
$\mathbf{m_{KM}}$	-	-	-	0/0.05	0/0.05	-	0/0.05
$\mathbf{m_{CaH}}$	-	-	-	0/0.01	0/0.01	-	-
